# A qualitative study of the barriers to commissioning social and therapeutic horticulture in mental health care

**DOI:** 10.1186/s12889-024-18621-8

**Published:** 2024-04-29

**Authors:** Carly J. Wood, Georgina Morton, Kathryn Rossiter, Becs Baumber, Rachel E. Bragg

**Affiliations:** 1 School of Sport, Rehabilitation and Exercise Sciences, University of Essex, Colchester, CO43SQ UK; 2 Thrive, Geoffrey Udall Centre, Beech Hill, Reading, RG72AT UK; 3 Rachel Bragg Consultancy, Hereford, HR11GZ UK

**Keywords:** Social and therapeutic horticulture (STH), Nature-based intervention (NBI), Green social prescribing (GSP), Mental illness, Mental ill-health, Commissioning, Mental health, Health care

## Abstract

**Background:**

Social and Therapeutic Horticulture (STH) is a process where trained practitioners work with plants and people to improve an individual’s physical and psychological health, communication and thinking skills. Evidence suggests that STH can support individuals with mental ill-health, however, current commissioning of STH within mental health care is limited. This study aimed to understand the barriers to commissioning STH in mental health care and to identify potential solutions to barriers, to support more widespread availability of services.

**Methods:**

Individuals with a role in mental health care commissioning from across the UK were invited to take part in semi-structured interviews via zoom. Interviews explored factors influencing the mental health services they commission or refer to, their perception of the role of STH in mental health care and the barriers to commissioning STH, together with potential solutions to any barriers identified.

**Results:**

Commissioners identified a lack of knowledge of STH and evidence of its effectiveness, and a culture which prioritises traditional medical models, as barriers to commissioning. Challenges for STH providers in responding to large-scale commissioning requirements were also highlighted as a barrier.

**Conclusions:**

To upscale commissioning of STH in mental health care, STH interventions need to be embedded within NHS priorities and information on STH services and their effectiveness needs to be easily accessible to practitioners. The sector should also be supported in working collaboratively to enable commissioning of services at scale.

**Supplementary Information:**

The online version contains supplementary material available at 10.1186/s12889-024-18621-8.

## Background

Mental health is defined as “a state of well-being in which every individual realises their own potential, can cope with the normal stresses of life, can work productively and fruitfully, and is able to make a contribution to her or his community” [1]. Mental illness or mental ill-health is characterized by a clinically significant disturbance in an individual’s cognition, emotional regulation, or behaviour, that is associated with distress or impairment in important areas of functioning, such as work, daily activities, or personal relationships [2]. The NHS identify five mental health levels which capture both mental health and mental illness on a continuum [3], moving from Level 0, where a person can take their own decision to independently improve their mental health and wellbeing, through to level 4, a person who is experiencing acute mental health crisis or who has a long-term serious mental illness. It is expected that people move through the different mental health levels either on a recovery pathway, or during times when mental health worsens and an increased level of intervention is required.

Common treatment approaches for mental health levels 2 and above include medication and psychological therapies [4]. However, medications such as anti-depressants may only provide significant positive effects for severe depression (level 4) [5] and have side effects or withdrawal symptoms [6]. Recent clinical trials also indicate that the efficacy of psychological therapies such as cognitive behavioral therapy (CBT) has diminished [7] whilst long waiting lists [4], can leave individuals without treatment for significant periods of time.

Social and Therapeutic Horticulture (STH), a process where trained practitioners work with plants and people to improve an individual’s physical and psychological health, communication and thinking skills [8], is one type of nature-based intervention (NBI) that is used to support individuals with mental ill-health. Although often used as an umbrella term for all gardening activities that target health and wellbeing, STH represents more targeted gardening activities that support individuals at mental health levels 2 and 3, typically provided by the voluntary, community and social enterprise (VCSE) sector. More specialised provision (termed ‘horticultural therapy’) for level 4 mental health needs where patients are typically in hospital or in-patient settings, and less specialised social gardening for level 1 needs is also available.

To date, multiple systematic reviews and meta-analyses have been conducted on the benefits of gardening and STH activities, reporting reductions in symptoms of depression and anxiety, reduced stress and mood disturbances, and improved quality of life, life satisfaction and community belonging for a range of groups, including the general public, patients with a range of physical illnesses, those with poor mental health, symptoms or diagnoses of mental illness [9–15]. In a recent review of randomised controlled trials (RCTs), Briggs, Morris, and Rees [16] revealed an overall positive effect of STH interventions for depression and wellbeing, with half of the included studies involving individuals with a diagnosis or symptoms of mental illness. Despite the positive findings from this review, much of the existing evidence of the benefits of STH is focused on gardening and gardening activities for individuals at mental health level 0 and 1. There are fewer published scientific studies specifically focusing on individuals at mental health levels 2–4 who would need to be referred to STH interventions. Furthermore, most published studies use pre-post design methodologies without control groups, and incorporate a variety of outcome measures, thus making it difficult to combine findings across studies.

Despite evidence of the benefits of STH for a range of populations, and an increased interest from the Government and NHS [17] in the health and wellbeing benefits of engaging with nature, referrals to STH are not widespread from the NHS or within social prescribing schemes [18]. Current commissioning of NBI such as STH are primarily from the local authority, social services, self-referrals, special education, and Community Mental Health Teams [18, 19], rather than routinely from general practitioners (GPs) or other clinicians. The NHS commissioning cycle involves a continual process of (i) strategic planning (to identify needs, review provisions and decide priorities); (ii) procurement of services and (iii) monitoring and evaluation [20]. Given that the NBIs have been highlighted as a key priority for improving mental health [17], wider commissioning might be expected. However, Shaw et al. [21] highlighted that commissioning for long term conditions is labour intensive for commissioners, with the scale and intensity of the work often not being proportionate to the service gains. It was also reported that commissioners were less comfortable with the transactional elements of their role, such as decommissioning services or seeking new providers [21]. These factors might act as barriers to commissioning of STH for mental health.

There has recently been a fundamental shift in the way that the health and care system is organized in the UK. In July 2022, Integrated Care Systems (ICS) were given statutory status, with Integrated Care Boards (ICBs) being set up to take on the NHS planning functions previously held by clinical commissioning groups, enabling joined up working and partnerships between health and social care and VCSE organisations [22, 23]. It was hoped that this shift might result in increased commissioning of services based in VCSE sector, however, there also may be unique and unknown challenges experienced within this new structure. The aims of this study were therefore to (i) understand the barriers to commissioning STH in mental health care and (ii) identify potential solutions to these barriers to support more widespread availability of services.

## Methods

### Design

To understand barriers to commissioning STH in mental health care, a combined deductive and inductive qualitative approach was used [24]. Semi-structured interviews, a common qualitative method, were conducted to obtain in-depth information about the experiences and perspectives of individuals with a role in mental health care commissioning. The interviews were conducted by a research assistant trained in qualitative data collection techniques and analysis.

### Study context

Potential participants were identified by the research team and members of the Therapeutic Horticulture Stakeholder Group (THSG), a group established by Natural England in March 2022 with support from The National Academy of Social Prescribing (NASP), to explore how to grow the Therapeutic Horticulture offer and to support the scaling up of Green Social Prescribing (GSP) [25]. The group (currently chaired and convened by Thrive with support from Natural England) brings together leading organisations and professionals in this field with representation from Natural England, The National Academy of Social Prescribing (NASP), NHS England, academics, health care professionals, and organisations who support the provision of STH. With permission, THSG members provided the research team with the email addresses for individuals with a role in mental health care commissioning who might be interested in taking part in the research.

### Recruitment

Individuals were purposively selected for participation in the research based on their job role to ensure representation across mental health ‘commissioning’ roles, including individuals who refer individual patients to mental health services through to those in senior positions with responsibility for commissioning regional mental health services. Participants were also selected to incorporate the perspectives of individuals both with and without experience of commissioning or referring to STH interventions and from across multiple regions of the UK. All potential participants were contacted via email by a member of the research team and provided with information about the study via a participant information sheet. Potential participants also shared information about the research with their colleagues who were also invited to take part in the study. A combined purposive and snowballing sampling approach was therefore used, two sampling techniques that are commonly combined [26]. In total 22 participants were invited to participate in the study.

Prior to participation in the research, participants were sent the definition of STH [8] and the mental health levels [3] to aid discussion of the role of STH in mental health care and to ensure consistency in their understanding of both STH and the NHS mental health levels. All participants provided informed consent prior to participation in the study and reconfirmed their consent at the start of the interview. Ethical approval was granted by Ethics Sub-committee 2 at the University of Essex (ETH2223-0519). Regulations regarding data management and storage were adhered to throughout the research.

### Characteristics of participants

Nine participants provided consent to take part in an online semi-structured interview via zoom, including five males and four females. Participants were from a range of roles related to mental health care, with some participants referring individual service users to local services and others commissioning services for an entire region. Participants were a link worker, a GP, a consultant psychiatrist, a clinical psychologist, a commissioner of mental health services for children and young people, a community mental health team project manager, director of adult mental health, head of commissioning and policy, and a mental health programme lead. Most participants (*n* = 5) reported having a general awareness of GSP and NBI but no expertise in STH, whilst the remaining participants (*n* = 4) reported extensively researching STH and commissioning or supporting STH-type services. Participants were from multiple regions across the UK (with two participants spanning two regions), including Essex (*n* = 4), Suffolk (*n* = 1), Lancashire (*n* = 1), South Cumbria (*n* = 2), Somerset (*n* = 1), Kent (*n* = 1) and Manchester (*n* = 1).

### Interviews

Semi-structured interviews were conducted between February-April 2023. Interviews were conducted electronically in a private space at the participants and researcher’s place of work or in their homes. Interviews lasted between 17 min and 47 min, with this variation in duration resulting from the mixed experiences of STH amongst participants. Interviews were recorded using Zoom software and automated transcripts downloaded, checked, and corrected by the research assistant prior to analysis. All participants were asked about their job role, the factors that influence the mental health services they commission or refer to, their perception of the role of STH and the barriers to commissioning STH, together with potential solutions to any barriers identified. The topic guide used in the interviews is included in Appendix 1. This guide was developed by the authors, in line with the study aims, with feedback provided by the THSG to refine the final interview guide.

### Analysis

Data were managed and coded using NVivo software version 12 (QSR International Pty Ltd., Doncaster, Australia, 2018). Transcripts were coded using reflexive thematic analysis, following the phases of Braun and Clarke [27, 28]. Initially, two interview transcripts were coded independently by two authors (CJW, GM) and following discussion, a coding framework was developed and used to code the remaining transcripts. The coding framework was revised as coding continued. Themes were actively produced through exploration of the data and codes, and subsequent discussions between the wider research team.

As data analysis progressed and themes developed, the researchers discussed their own assumptions of the codes and themes. The researcher with the least experience in the mental health benefits of STH carried out the primary analysis to ensure that there was the least bias in the coding of the data. In the final stage of the analysis, four overarching themes were identified, each of which are described in detail below and include funding and workforce (theme 1), commissioning culture (theme 2), knowledge of STH (theme 3) and evidence of effectiveness (theme 4). Within these themes both the barriers to commissioning STH in mental health care (aim 1) and potential solutions to the barriers raised (aim 2) are discussed.

## Results

### Theme one: funding and workforce

A lack of funding available for mental healthcare in the NHS was referred to as a key challenge for commissioning by most participants. Participants referred to a reduction in investment in mental health services over the last decade and reflected that a consequence of the reduced investment was that the NHS was “*trying to do more with less*”. Commissioning decisions were therefore suggested as being based around what can be delivered given the finances available and ensuring that commissioned services are “*cost effective*”. Commissioners were reported as being left in a position where they must commission based on what they can afford to provide rather than based on what they perceive to be best for their population.

In relation to STH specifically, most participants reported that the limited budgets available for mental health care result in sustainable and longer-term funding being a persistent problem for VCSE organisations. This was thought to result in the short-lived nature of STH services and high staff turnover. The lack of consistency in the offer across regions was also thought to compound this problem. Participants commented on the need to commission services that can cater for the entire population for which they commission, with “*pockets of services”* making it difficult to do so. These “*pockets of services*” were deemed as not always being in areas where they were most needed, being less cost-effective, not being accessible for all and potentially requiring transportation to reach, which may pose financial issues for both individuals and organisations.

“They don’t want to be having just one project in one corner of their patch. They want to be able to say we’re doing this across the whole county.” (General Practitioner)

It was suggested that partnership working within the VCSE sector would enable a more consistent offer and a larger “*footprint”* across regions, which would support access to larger funding streams, the growth of smaller VCSE organisations and subsequently wider scale commissioning of services.

Similar to a shortage of funding, most participants referred to a lack of workforce and resources within mental health services. One participant referred to a *“revolving door of personnel”*, resulting in continued staff shortages, whilst another referred to the constant juggling of resources. These issues were suggested to be a barrier to commissioning of new services such as STH.

“...new idea is...something they've not got time for as they’re so bogged down, it’s just kind of surviving day to day really. It’s almost too much to start thinking about something new, like a new nature-based therapy group.” (Clinical Psychologist)

One participant reported that the loss (and lack of replacement) of staff in particular roles essential to furthering the NHS Trusts green plans and working with the VCSE sector, further limited commissioning of STH. It was felt that embedding sustainability roles into Trusts would save money and that having directors and ‘champions’ who have a personal interest in sustainability, would help to influence commissioning boards and push the sustainability and STH agenda forward. However, the association between sustainability roles and commissioning of STH was only made by one participant, making it unclear to what extent these are reliant upon each other.

It was also reflected by one participant that the underfunding and understaffing issues within the NHS might present a key opportunity for the VCSE sector to assist in providing mental health care if it is given the chance, with another participant referring to the *“missed opportunity”* within their Trust to use small pots of funding to support the VCSE sector.

### Theme two: commissioning culture

Several participants suggested that commissioned mental health services are driven by national requirements set out by the NHS and in the long-term plan [29] and that services such as STH are *“not really embedded in national must dos…”* Rather than facilitating a holistic approach to commissioning, the NHS guidelines (combined with the underfunding and under-resourcing of mental health care) were thought to limit the capacity of commissioners to allocate funding for services within the VCSE sector. Participants emphasised the need to see “*green initiatives*”, “*efforts*” and “*schemes*” within these national plans to support the commissioning of STH.

The commissioning culture of a *“focus on reactive treatments rather than prevention”*, was also reflected as a barrier to commissioning STH, with several participants discussing prevention of mental illness in relation to STH. Traditional approaches and therapies (i.e., talking/cognitive and drug therapies) were suggested as being prioritised, with a need to shift towards more preventative and holistic treatment in order for services like STH to be fully embedded.

“I think what we’re trying to do is stop the knee-jerk reaction to ‘we have to plug a gap over here’ and thinking about it more creatively and that’s what we’re trying to do. But it’s a big shift for the system, and it’s really easy to just keep throwing money at something that is a traditional approach to fixing something”. (Commissioner of mental health services for Children and Young People)

Some participants also suggested that STH should be embedded at every level of mental health care, allowing patients at all levels of mental health need to be referred to VCSE sector services and via a number of different referral pathways.

“It’s obvious, you build it in at all levels of referral...before GP, at GP, at IAPT [Increasing Access to Psychological Therapies], at secondary care. You just open the doors, and it would be successful. Reduce the demand on the NHS” (Consultant Psychiatrist).

It was felt that this approach would support individuals in accessing STH services, but that in order for it to be embedded at every level there would need to be “buy-in” from commissioners.

### Theme three: knowledge of STH

Whilst all participants felt that there was a role for STH in mental health care, a lack of knowledge of STH by individuals with roles in referrals and commissioning was reported as a barrier to commissioning. While some participants within the study demonstrated or reported good knowledge of what STH is, the services available, and the range of mental and physical health benefits it could provide, this was cited as not being the case for all individuals within their organisations, where there was a mixture of different levels of knowledge. Some study participants also reported (or demonstrated) that they personally had limited knowledge of what STH is, who it is for and/or the evidence base surrounding the health benefits. There were some perceptions that STH would only appeal to certain groups and that it could only play a role in mental ill-health prevention or maintenance rather than in treatment, which contradicts the evidence supporting the use of STH in health care.

“This type of activity probably appeals to people in a particular demographic...I'm not necessarily convinced that people in their twenties and thirties would think of that as a go-to for leisure, pleasure, or seeing that as something that would benefit them..”. (Head of Commissioning and Policy)

The limited knowledge of STH was largely attributed to a lack of available information from providers of STH about the benefits of their services, who they are targeting, and how risk is managed. Most participants reported not receiving information or it not being readily available or easily accessible amongst the large volume of information that commissioners already receive. Some participants also referenced the need for the VCSE sector to promote or ‘champion’ their services and directly approach the NHS to highlight what they were doing within the community and to identify how this might align with ongoing NHS agendas.

“We need to be able to understand what the offer is, and it’s not always clear what community assets are available, and so I think the sector could do a better job for sure of collating those offers. But we need to understand what it is, what the needs are, what the value is, and how we can support it in a financially challenged environment.” (Community Mental Health Team Project Manager)

Overall, participants felt that greater and more effective sharing of information on STH and communication with commissioners was needed for STH to be commissioned more widely.

“Why are we not doing it? We don’t really know what they’re doing”. (Link Worker)

This was felt to be particularly important given that commissioners do not typically get training in STH.

### Theme four: evidence of effectiveness

Evidence was highlighted as a factor influencing commissioning by all participants involved in the study, but to varying degrees and in varying contexts, perhaps reflecting differences in the knowledge of participants. Evidence of the effectiveness of STH was perceived by several participants as lacking in quantity and quality, with some reference to the need for high quality studies. Some participants also referred to a lack of awareness of evidence of the benefits of STH, in line with a lack of knowledge of STH broadly (Theme 3). However, one participant with extensive experience of STH, said that lack of evidence in relation to the benefits of STH was not the issue but rather a lack of evidence of how STH can “*structurally work within government commissioned services*”, alluding to potential difficulties in embedding services such as STH throughout the healthcare system.

Some participants also referred to the differences in evidence between levels of mental health need and how it was not necessarily effective for all mental health conditions, with one participant stating that it is not a *“universal panacea”*. One participant referred to the evidence of STH for severe and enduring mental illness and that whilst there was evidence to support its use, it was not widely publicised. Participants felt that evidence of the benefits of STH needed to be shared widely, easy to access and regularly updated.

“..the longer you work as a doctor, the less you become an academic because you become a clinician, so it’s less easy to access all that information. So, it’s a bit difficult to kind of prove to people that there is some decent evidence.” (Consultant Psychiatrist)

Several participants also referred to key performance indicators that the NHS are measured against and the need for STH services to have measurable outcomes that align with these indicators, for example the Warwick Edinburgh Mental Wellbeing Scale. A number of participants also commented that these outcomes should be focused on the effect STH has had on the individual patient, instead of statistics like waiting, access and discharge rates, which do not identify whether the patients’ condition has improved. However, there were also contradictory points highlighting that commissioning decisions were typically based around referral and discharge rates, the longer-term impacts on the healthcare system, and cost savings for the NHS, with these statistics being easier to examine than the impacts on patients.

As a result of challenges over measurement of outcomes and impact, some participants suggested changes to the ways that STH providers collect and provide evidence. Participants recommended that the sector focuses on providing qualitative evidence such as “*case studies*”, “*vignettes*” or “*user experience voices*” that tell “*the positive story*” of the impact their service has for the individual.

“...Health has a high bar for reporting, and we need to be able to prove that something has had an impact.. We can’t do the same thing really, with some of the green investments that we make. And so, I think we need to understand how we can evidence the impact it’s had, and it doesn’t always need to be data driven ...There are number-driven discussions, or data driven discussions. What is missing in that room is the patient’s story and the impact. And I think that’s where the third sector could really help us bring this to life.” (Community Mental Health Team Project Manager)

However, this type of evidence was acknowledged as being difficult to accomplish and often limited by the infrastructure of the organisations who may not have the capacity to collect this information. One participant suggested that if this evidence was available, the use of a video to demonstrate the impact on patients might be a technique that would *“sell”* the service to commissioners.

## Discussion

The aims of this study were to (i) understand the barriers to commissioning STH in mental health care and (ii) identify potential solutions to these barriers to support more widespread commissioning of STH services. The key themes that were produced from the data were issues around funding and workforce which prevented widespread commissioning of STH, a commissioning culture which makes it difficult to commission ‘non-traditional’ treatments, a lack of knowledge of what STH is and how it can be used, the services available, and a lack of [awareness of] evidence to support its effectiveness. There were a number of suggestions as to how these barriers could be overcome, most of which are likely to require systems-level change by both the NHS and VCSE sector.

In relation to funding and workforce, the continued reductions in funding for mental health care were identified as a key barrier to commissioning STH. This finding is mirrored in the recent evaluation of the Government’s GSP pilot, which identified unstable short-term funding and lack of system level support for the sector as a barrier to embedding GSP within statutory systems [22]. Furthermore, the recently established, ICBs, which were designed to support greater partnership working with the VCSE sector, have been asked to make a further 30% reduction in their running costs [30]. As a result, funding and resources for mental health services are likely to become even more stretched, further restricting commissioning of new services.

In the UK most NBIs, including STH, sit within the VCSE sector and are typically delivered by small-scale providers, allowing for a more bespoke, person-centred service [22, 31]. However, this approach makes it difficult for STH providers to respond to large-scale commissioning requirements and combined with the funding and resources issue, is likely to result in commissioners continuing to consider STH as a less viable option for mental health care. Thus, it is essential that STH providers work in partnership to demonstrate the ‘offer’ for services they can provide on a regional scale [32, 33]. This collaborative approach could be supported and facilitated through the use of regional nature-based VCSE networks such as the Norfolk Green Care Network [34] and the Reading Green Wellbeing Network [35]. These networks can promote partnership working between providers, become potential commissioning hubs and could enable providers to work together to apply for larger funding opportunities. Voluntary networks such as these could also help ICBs proactively engage with VCSEs but would need investment and support at the system-wide level to ensure sustainability.

Commissioning culture within the health service was also identified as a key barrier to commissioning of STH. Despite a commitment to increase use of personalised care, social prescribing, and community centred approaches for health and wellbeing across multiple Government and health organisations [36, 37], the NHS long plan [29], which outlines the key priorities from 2019 to 2024, does not embed the use of these approaches as priorities. Instead, it prioritises helping people to get easier access to therapy for common mental disorders such as anxiety and depression; despite evidence to suggest diminishing effectiveness over time and poor outcomes for some groups [7]. Without community-based approaches being embedded within national plans, participants felt they had limited capacity to commission the VCSE sector.

The recently published NHS major conditions strategy case for change and strategic framework [38] calls for a focus on integrated working with community-based partners as part of the future long term conditions strategy, and a commitment to accelerating research to understand how mental, physical, and social conditions interlink and how they can be treated. Given that services such as STH can target mental, physical, and social needs simultaneously [39], it is possible that this focus may result in increased use of holistic services such as STH. However, until the full long-term conditions strategy is released, it is unclear how these approaches will be embedded and prioritised. As highlighted by participants, for interventions such as STH to be successful, they need to be embedded at every level of mental health care, allowing multiple entry points into the VCSE sector. The trend for prioritisation of traditional approaches to mental health care, as also reported by Shanahan et al. [40] and Tambayah et al. [41], alongside the suggested reluctance of commissioners in decommissioning services and seeking new providers [21], also needs to be overcome to promote greater variability in treatment options.

Lack of knowledge and awareness of STH, in a variety of contexts, was highlighted as a key barrier to service commissioning. There were some perceptions that STH would not appeal to all individuals or that it was not suitable for particular groups, for example younger people. A lack of knowledge about what STH interventions entail and the level of mental health need they can be appropriate for, was also highlighted by participants, with some interviewees referring to STH as solely a preventative health measure as opposed to a treatment option for acute and chronic mental illness. Furthermore, a lack of knowledge and awareness of what STH provision is available was identified as a barrier to commissioning. Lack of knowledge of local services has also been identified as a barrier to commissioning NBIs via GSP [22] and for commissioning STH by clinicians [42]. As commissioning of new services requires significant partnership working between both commissioners and service providers [21], this lack of awareness of what STH services are available locally is likely to be problematic.

Shanahan et al. [40] and Fixsen and Barrett [43] highlighted that referral and commissioning of NBI is influenced by the knowledge and interest of the GP, termed *“GP buy-in”*. Thus, individuals may not be offered interventions such as STH unless their health care provider has a particular interest in, knowledge of, or belief in its value. This need for *‘practitioner buy-in*’ is not aligned with traditional approaches where treatments are prescribed as ‘normal practice’ regardless of whether the practitioner has a particular interest in the approach. Providing a means by which practitioners can easily access information about STH services, such as regional or national directories of STH services, which enable identification of interventions across the UK and detail what they involve and who they are for, may facilitate increased awareness, knowledge and ‘*buy- in’* of STH interventions. However, any directory would need to be fully embedded in healthcare treatment, referral, and commissioning systems.

An interesting observation that emerged from the data was also the tendency of participants to refer to STH as green *“schemes”*, *“therapies”* or *“initiatives”*, indicating a perception that all nature-based activities are equivalent as reported by Sempik, Hine and Wilcox [44]. This is problematic and is likely to compound issues around what types of STH services are appropriate for different levels of need. To address this barrier, a framework for aligning STH provision with the NHS’ five mental health levels has been produced, identifying what types of activities, support, evaluation, and quality assurance are needed at each level, along with examples of beneficiaries across the UK [45]. To support partnership working, increased understanding and commissioning of STH, this framework should be adopted widely by both the health care sector and STH organisations and utilised in the suggested service directory.

Evidence of the effectiveness of STH was mentioned by all study participants as a factor that influences commissioning. Whilst some referred to a lack of awareness and publicisation of the evidence, as echoed in Tambayah et al. [41], others reported a lack in quality and quantity, or a lack of evidence for specific mental health levels or conditions. For individuals at mental health levels 0 and 1, there are a range of systematic reviews and meta-analyses demonstrating the benefits of gardening activities [11, 12, 14, 15]. There are also numerous studies and reviews reporting the benefits for STH for individuals with symptoms of mental illness or diagnosed mental illness, aligning with mental health levels 2–4. However, in many cases this data is combined with data from individuals without mental ill-health, or for a range of mental health disorders [13, 16, 46], making it more difficult to isolate the evidence for specific conditions and those who require mental health intervention. Whilst studies focused on individuals at levels 2–4 with mild to severe mental illness have demonstrated positive effects for depression, wellbeing, quality of life and activities of daily living [16, 47], many studies fail to incorporate comparison groups or randomisation procedures. To further enhance the evidence base, well-designed, high quality RCTs are therefore needed, along with sufficient funding to support this level of scientific evaluation.

Whilst there is undoubtedly room for high quality RCTs to further advance the STH evidence base, other accepted interventions in health and policy fields in the UK have not been based on RCT evidence [48]. There is also a wealth of quantitative and qualitative evidence from the scientific and VCSE sector advocating the effectiveness of STH, much of which utilises measurable outcomes and describes the impact on the patient (as suggested by the study participants). Furthermore, an independent report by the Kings Fund [48] suggested that gardening-based interventions can have numerous benefits for individuals as an adjunct to their existing mental health treatment, whilst the Wildlife Trusts [49] demonstrated significant cost savings to the NHS if they were to invest in a ‘natural’ health service, with an estimated an annual cost of £534.1 million per year for delivery against a gross annual cost saving of £635.6 million. Thus, whilst there is need to strengthen the evidence base in specific areas, there is clear evidence of the potential benefit of NBIs such as STH to the health care system and patients. Furthermore, Wye et al. [50] reported that commissioners experience multiple barriers to using academic research to inform commissioning. As a result, they often utilise NICE guidelines, local evaluations, local clinicians’ knowledge, and service users experiences to inform their commissioning decisions. To support commissioning of STH, existing evidence and knowledge should be integrated into mental health care policy and practice, NICE guidelines, and be more clearly publicised and communicated to commissioners via effective dissemination methods such as infographics and via professional journals aimed at commissioners.

The findings of this study present the perspectives of nine individuals, from a range of commissioning roles and regions across the UK. However, the full range of barriers experienced by individuals with roles in mental health care commissioning may not have been captured. Further research in this field should aim to incorporate the perspectives of individuals involved in the development of mental health policy and NHS senior leaders who have a direct influence on funding decisions, to understand the barriers to prioritising approaches such as STH at a national level. It should also prioritise high quality RCTs for mental health levels 2–4 and for specific conditions, to develop a clearer and more focused evidence base to support commissioning of STH in mental healthcare. The potential solutions to the commissioning barriers highlighted in this research should also be actioned by individuals in health and VCSE sectors to further support the growth and commissioning of STH. This is essential for ensuring a more sustainable mental health system whereby service users can access support when it is needed.

## Conclusions

Overall, the findings of this study highlight a range of barriers to the commissioning of STH, including a commissioning culture which priorities traditional medical models, a lack of knowledge of STH broadly (including the services available, levels of mental health need it can cater for and the existing evidence of its effectiveness, particularly for specific mental health conditions), and the challenges for STH providers in responding to large-scale commissioning requirements. To support commissioning of STH in mental health care, the VCSE sector should be supported in developing higher quality evaluation methodology accepted by the NHS and in working collaboratively to enable commissioning of services at scale. Information on STH services and their effectiveness also needs to be easily accessible to practitioners, and STH interventions should be fully embedded within NHS priorities to enable a more holistic health care approach, which has the potential to improve patient outcomes, reduce the strain on mental health services and result in considerable cost savings.

### Supplementary Information

**Supplementary Material 1.**

## Data Availability

The datasets generated during the current study are available in the REShare repository, with restricted access via https://reshare.ukdataservice.ac.uk/856812/.

## Abbreviations

CBT: Cognitive Behavioural Therapy
GP: General Practitioner
GSP: Green Social Prescribing
ICB: Integrated Care Board
ICS: Integrated Care System
NBI: Nature-based Intervention
NHS: National Health Service
RCT: Randomised Controlled Trial
STH: Social and Therapeutic Horticulture
VCSE: Voluntary, Community and Social Enterprise
