# Supplementary material for: A qualitative study of the barriers to commissioning social and therapeutic horticulture in mental health care
Source: BMC Public Health. 2024 Apr 29;24:1197. doi: 10.1186/s12889-024-18621-8 (PMC11059742; doi:10.1186/s12889-024-18621-8)
Supplement: Supplementary file 1 — Supplementary Material 1. [file 12889_2024_18621_MOESM1_ESM.docx]

**Appendix One: Semi- Structure Interview Guide**

**Introductions and Welcome to session**

**Participant Job Role**

Could you tell me about your job role and what role you play in commissioning of mental health services?

Based on the five levels of mental health shared prior to the interview, which level of mental health do you commission services for?

**Factors influencing commissioning of mental health services.**

In your experience, what are the key factors that influence which mental health services are commissioned within your area?

Are any of these factors more influential than others? If so, which ones and why?

In your experience, do the factors influencing commissioning of mental health services vary between regions? If so, could you give examples of this variation.

**Social and Therapeutic Horticulture in Mental Healthcare**

What is your understanding of social and therapeutic horticulture (STH), and the mental health benefits it can provide?

Do you see a role for STH in mental healthcare? If so, what role do you see STH playing? If not, could you explain your reasons for this?

What level of mental health need do you believe STH can cater for? Please explain your answer.

Do you currently commission or know of STH services that are commissioned in your area? If so, could you tell us more about these services and how they are commissioned/referred to?

What is your understanding of the role that social prescribing could play in referring individuals with mental ill health to STH?

**Barriers to commissioning social and therapeutic horticulture**.

Based on your experience, what are the challenges or barriers to commissioning of STH services in your area?

Do you consider these challenges/barriers to be unique to your area and/or to STH? If so, why?

Are there any solutions or strategies that you believe may help to overcome these barriers/challenges? If so, what are they?

**Final Question**

Is there anything you want to tell me that I have not asked?

**Close the session.**
